# N6-methyladenosine-modified circ_0000337 sustains bortezomib resistance in multiple myeloma by regulating DNA repair

**DOI:** 10.3389/fcell.2024.1383232

**Published:** 2024-03-22

**Authors:** Siyi Jiang, Lili Gao, Jian Li, Fangrong Zhang, Yanan Zhang, Jing Liu

**Affiliations:** ^1^ Department of Hematology, The Third Xiangya Hospital of Central South University, Changsha, China; ^2^ Jinan Hospital of Integrated Chinese and Western Medicine, Jinan, China; ^3^ Department of Blood Transfusion, The Third Xiangya Hospital of Central South University, Changsha, China

**Keywords:** multiple myeloma, bortezomib resistance, M6A, circ_0000337, DNA2

## Abstract

Studies have shown that bortezomib resistance in multiple myeloma (MM) is mediated by the abnormalities of various molecules and microenvironments. Exploring these resistance mechanisms will improve the therapeutic efficacy of bortezomib. In this study, bone marrow tissues from three patients with MM, both sensitive and resistant to bortezomib, were collected for circRNA high-throughput sequencing analysis. The relationship between circ_0000337, miR-98-5p, and target gene DNA2 was analyzed by luciferase detection and verified by RT-qPCR. We first found that circ_0000337 was significantly upregulated in bortezomib-resistant MM tissues and cells, and overexpression of circ_0000337 could promote bortezomib resistance in MM cells. circ_0000337 may act as a miR-98-5p sponge to upregulate DNA2 expression, regulate DNA damage repair, and induce bortezomib resistance. Furthermore, it was determined that the increased circ_0000337 level in bortezomib-resistant cells was due to an increased N6-methyladenosine (m6A) level, resulting in enhanced RNA stability. In conclusion, the m6A level of circ_0000337 and its regulation may be a new and potential therapeutic target for overcoming bortezomib resistance in MM.

## 1 Introduction

Multiple myeloma (MM) is a malignant tumor with abnormal plasma cell proliferation (Huber et al., 2023). According to the American Cancer Society estimates, the number of new cancer cases is 35,780, and the deaths are 12,540 in MM (Siegel et al., 2024). The clinical manifestations of this disease are varied, including bone pain, anemia, renal insufficiency, infection, amyloid change, etc., which is easy to misdiagnose (Shen et al., 2021). Chemotherapy and stem cell transplantation are the main treatment methods of MM; hematopoietic stem cell transplantation is not only costly but also prone to recurrence (Tacchetti et al., 2020; Beksac and Hayden, 2023). Bortezomib is a reversible inhibitor of 26S proteasome chymotrypsin-like activity in mammalian cells and is currently a first-line drug for multiple myeloma (Chroma et al., 2022). Bortezomib has a good therapeutic effect and can prolong the survival of MM patients. However, bortezomib resistance often occurs in the course of use, which affects the therapeutic effect of MM (Liu et al., 2021). Although several mechanisms of bortezomib resistance have been reported, mainly related to genetic alterations in multiple myeloma, the exact cause remains unknown (Chroma et al., 2022; Aziz et al., 2023; Ochiai et al., 2023). Therefore, it is imperative to decipher the underlying molecular mechanisms of bortezomib resistance and to explore new strategies to counteract it.

Circular RNAs (circRNAs) are closed non-coding RNAs formed by reverse splicing (Xue et al., 2021; Mirazimi et al., 2023). circRNAs play an important role in diseases, especially in cancer, and have unique advantages in disease diagnosis and treatment strategy development (Wang, 2022; Yu et al., 2023). Importantly, in the process of tumor progression, many circRNAs are involved in proliferation, differentiation, invasion, and metastasis (Fontemaggi et al., 2021; Qadir et al., 2023). Recent research has shown that circ_0000190 can inhibit the development of multiple myeloma by promoting MAPK4 expression by spongifying miR-767-5p (Xiang et al., 2022). It was found that circHNRNPU was one of the most differentially expressed circRNAs in MM. circHNRNPU is secreted by MM cells and can encode a protein (circHNRNPU_603aa). Over-expressed circHNRNPU_603aa can promote MM cell proliferation (Tang et al., 2022). However, the role of circRNA in bortezomib-resistant MM remains unclear.

N6 methyladenosine (m^6^A) methylation is the most common internal modification of mRNA and non-coding RNA in eukaryon (Zhuang et al., 2023). m^6^A modulation is performed by m^6^A methyltransferases (writers), demethylases (erasers), and methylated readers in complex functions involving a large number of pathophysiological processes (Uzonyi et al., 2023). Extensive studies have shown that m^6^A has a regulatory role in a variety of tumors and has an impact on tumorigenesis, metastasis, and drug resistance. HNRNPA2B1, an m^6^A reader, was found to be elevated in patients with MM and inversely associated with a good prognosis. HNRNPA2B1 recognizes the m^6^A site of ILF3, enhances the stability of ILF3 mRNA transcripts, and induces cell proliferation (Jiang et al., 2021). Heat shock factor 1 (HSF1) is a regulatory target of FTO-mediated m^6^A modification. FTO targets HSF1/HSPs in a YthDF2-dependent manner, significantly promoting MM cell proliferation, Invasion, and metastasis (Xu et al., 2022). However, the role of m6A modification in bortezomib-resistant MM remains unclear, and the role of m6A-modified circRNA in bortezomib-resistant MM also warrants further investigation.

In this study, we discovered that circ_0000337 is upregulated in bortezomib-resistant MM and is necessary for maintaining bortezomib resistance. circ_0000337 can act as a sponge for miR-98-5p to upregulate DNA2 expression, which regulates DNA damage repair and induces bortezomib resistance. Additionally, the increase in circ_0000337 levels in bortezomib-resistant cells was found to be due to the elevated m6A methylation of specific adenosines, resulting in enhanced RNA stability. This study uncovers a new mechanism for MM bortezomib resistance, suggesting that the m^6^A methylation level of circ_0000337 and its regulation may be a potential therapeutic target for bortezomib-resistant MM patients.

## 2 Materials and methods

### 2.1 Materials

Bortezomib (SB8980) and Cell Counting Kit-8 (CK04) were obtained from Solarbio (Beijing, China). Animal Total RNA Isolation Kit (B518621), AMV One-step RT-PCR Kit (B532441), Dual Luciferase Reporter Gene Assay Kit (E608001), and primer were purchased from Sangon Biotech (Shanghai) Co., Ltd. circ_0000337 high-expressed and knock-down plasmid were purchased from GENECHEM (Shanghai, China). Anti-DNA2 antibody (ab96488), Anti-GAPDH antibody (ab8245), Anti-YTHDF1 antibody (17479-1-AP), Anti-YTHDF2 antibody (24744-1-AP), Anti-METTL3 antibody (15073-1-AP), and Anti-FTO antibody (27226-1-AP) are available from Proteintech (United States). Actinomycin D is purchased from Merck KGaA (Germany). DMEM (SH30249.02), RPMI 1640 (SH30027.01), fetal bovine Serum (SH30084.03), penicillin streptomycin (SV30010), and trypsin (SH30042.01) were purchased from HyClone (United States). Serum-free cell freeze (C40100) is produced by NCM Biotech (Suzhou, China).

### 2.2 MM tissue samples

MM specimens were collected from the Third Xiangya Hospital of Central South University. According to the NCCN Clinical Practice Guidelines (Kumar et al., 2023), the patients were divided into bortezomib-resistant (*n* = 25) and bortezomib-sensitive (*n* = 25). Bortezomib resistance or sensitivity was defined in MM with or without recurrence at the time of bortezomib application. All specimens and related studies received written informed consent from the patients. Human ethics and consent to participate declarations for the use of bone marrow (BM) samples were granted by the Medical Ethics Committee of the Third Xiangya Hospital of Central South University (Approval No: 2021-S440), and all subjects signed informed consent forms that were designed in compliance with the Declaration of Helsinki.

### 2.3 Cell lines and cell culture

In this study, we utilized MM cells procured from Abiowell (U266, AW-CCH366), and Procell (RPMI8226 CL-0564). BTZ-resistant cell lines (U266-R and RPMI-8226-R) were provided by the Department of Hematology, Third Xiangya Hospital of Central South University. The cells were inoculated in RPMI 1640 medium supplemented with 10% bovine fetal bovine serum, 1% penicillin, and streptomycin and placed in a 5% CO_2_ sterile incubator at 37°C. BTZ-resistant cells were maintained in RPMI 1640 medium, as described above, containing 2 nM BTZ.

### 2.4 Preparation of RNA and PCR assay

Total RNAs were extracted from MM tissues and cells with a Total RNA Isolation Kit (B518621, Sangon Biotech). The expression of circRNA_0000337, miR-98-5p, and DNA2 mRNA were determined with AMV one-step RT-PCR Kit (B532441, Sangon Biotech) by ABI 7500 Real-Time PCR System (United States). The primers used in this study are listed in Table 1.

**TABLE 1:** Primers and RNA sequences used in this study.

Primer sequence		
hsa_circ_0000337	Forward	GAT​GCC​TTG​GGA​CTT​AGC​AA
	Reverse	CGG​GGA​GGT​TTC​ACA​CTT​TA
GAPDH	Forward	AAT​GGG​CAG​CCG​TTA​GGA​AA
	Reverse	GCG​CCC​AAT​ACG​ACC​AAA​TC
DNA2	Forward	TTT​GCC​ACT​GCC​TAC​CAG​AG
	Reverse	GAG​ATA​CTG​GCA​GGT​CAG​GC
hsa-miR-330-5p	Forward	AAC​AAG​TCT​CTG​GGC​CTG​TG
hsa-miR-578	Forward	AAC​CGG​CTT​CTT​GTG​CTC​T
hsa-miR-326	Forward	TTC​TCC​AAA​AGA​AAG​CAC​TTT​CTG
hsa-miR-98-5p	Forward	AAG​CGA​CCT​GAG​GTA​GTA​AGT​T
hsa-miR-198	Forward	AAC​AAG​GGT​CCA​GAG​GGG​A
hsa-miR-1178	Forward	AAC​ACG​CTT​GCT​CAC​TGT​TC
hsa-miR-1204	Forward	AAC​AAT​TCG​TGG​CCT​GGT​C
hsa-miR-155	Forward	AAC​ACG​CTT​AAT​GCT​AAT​CGT​GA

### 2.5 RNA pull-down assay

The biotin-labeled circ_0000337 probe was constructed from Sangon (China). Briefly, circ_0000337-overexpressed U266 and RPMI 8226 cells were lysed and incubated with a biotin-labeled circ_0000337 or oligo probe. Streptomycin-labeled magnetic beads were added for adsorption, and the RNA complex bound to the magnetic beads was eluted for RT-PCR assay.

### 2.6 CCK-8 assay

1 × 10^4^ cells/well were incubated in a 96-well plate for 24 h. The different concentration of bortezomib (0–20 nmol/L) was added and incubated with cells for 24 h. Then, 10 ul CCK-8 solution was added to a 96-well cell culture plate and incubated in a 37°C incubator for 2 h. The absorbance of each well was detected at 450 nm with a microplate spectrophotometer (PerkinElmer, United States).

### 2.7 Cell transfection

A lentivirus vector inserted with circ_0000337 cDNA was constructed by GENECHEM (Shanghai, China). The PcDNA3.1 vector contains a front circular frame and a back circular frame. MM cells were screened with antibiotics after infection with Lentivirus for 24 h. The surviving cells were identified by qRT-PCR. Mutations of each miRNA-binding site in the circ_0000337 were constructed using a Hieff Mut™ Site-Directed Mutagenesis Kit (Yeasen, Shanghai, China). The mutations were introduced in the circ_0000337-expressed vector and the luciferase reporter with the circ_0000337.

### 2.8 Luciferase assay

The 3′UTR fragment of wild-type (wt) or mutant (mut) circ_0000337 was inserted into the pMIR-REPORT™ vector (Sangon, China). 104 cells per well were cultured in 96-well plates for 24 h miR-98-5p or anti-miR-98-5p with pMIR-REPORT-circ_0000337 (Wt/Mut) was cotransfected into U266 and RPMI 8226 cells by Lipo8000™ Transfection Reagent (Beyotime, China) for 48 h. Finally, luciferase intensity was detected by a microplate spectrophotometer (PerkinElmer, United States).

### 2.9 Western blot

MM tissues or cells were lysed by RIPA buffer (solarbio, China) with 1 mM PMSF for 20 min. After centrifugation at 12,000 rpm for 15 min. Total protein was obtained from the supernatant. The concentration of total protein was detected by an Enhanced BCA Protein Assay Kit (Beyotime, China). Denatured protein (20 μg/lane) was run in SDS-PAGE and then transferred to a PVDF membrane. PVDF membrane was then blocked with 5% bovine serum albumin for 2 h, followed by adding anti-DNA2 antibody and anti-GAPDH antibody for 12 h. After the unbound primary antibody was fully removed by PBS washing, HRP-conjugated secondary antibody (Boster, China) was added and incubated for 1 h. After adding the ECL Substrate to the PVDF membrane, the fluorescence signal was monitored by a chemiluminescence imaging system (Jiapeng, China).

### 2.10 RNA immunoprecipitation

Anti-METTL3 antibody (15073-1-AP), Anti-METTL14 antibody (26158-1-AP) and m6A Monoclonal antibody (68055-1-Ig) are available from Proteintech (United States). rProtein A/G IP/Co-IP Kit (36421ES40) was obtained from Yeasen (China). In brief, MM cells transfected with circRNA_0000337 plasmid for 48 h. Then, the cells were lysed in RIP lysis buffer, and incubated overnight with with Protein A-Sepharose beads and primary antibody at 4°C. After washing the complexes bound to the beads, RNA was extracted using a column. The expression of circRNA_0000337 and miR-98-5p was determined by RT-qPCR analysis.

### 2.11 Bortezomib-resistant MM mouse model

10^7^ U266-R cells mixed with Matrigel (BD, United States) were injected subcutaneously into the ventral side of BALB/c nude mice. Once the tumor volume reached 100 mm^3^, lentivirus with si-circ_0000337 precursor was injected around the tumor site. Meanwhile, bortezomib (0.5 mg/kg) was injected intraperitoneally. The tumor volume was monitored daily. Upon completion of the experiment, mice were systemically anesthetized. Tumor tissues were obtained for RT-PCR, western blot, and immunohistochemical staining. The methods employed in this study strictly adhered to the guidelines and regulations concerning the ethical use of live vertebrates in research as outlined by the Third Xiangya Hospital of Central South University. Furthermore, all procedures were conducted in accordance with the ARRIVE guidelines for reporting experiments involving animals.

### 2.12 Statistical analysis

All quantitative data are expressed as mean ± SD. The significant differences were performed by SPSS 18.0 software for statistical analysis. The differences between groups were analyzed by one-way ANOVA and subsequent Tukey’s post-test. A *p*-value of less than 0.05 was considered statistically significant.

## 3 Results

### 3.1 The expression levels of circRNA_0000337 in bortezomib-resistant multiple myeloma

**FIGURE 1:**
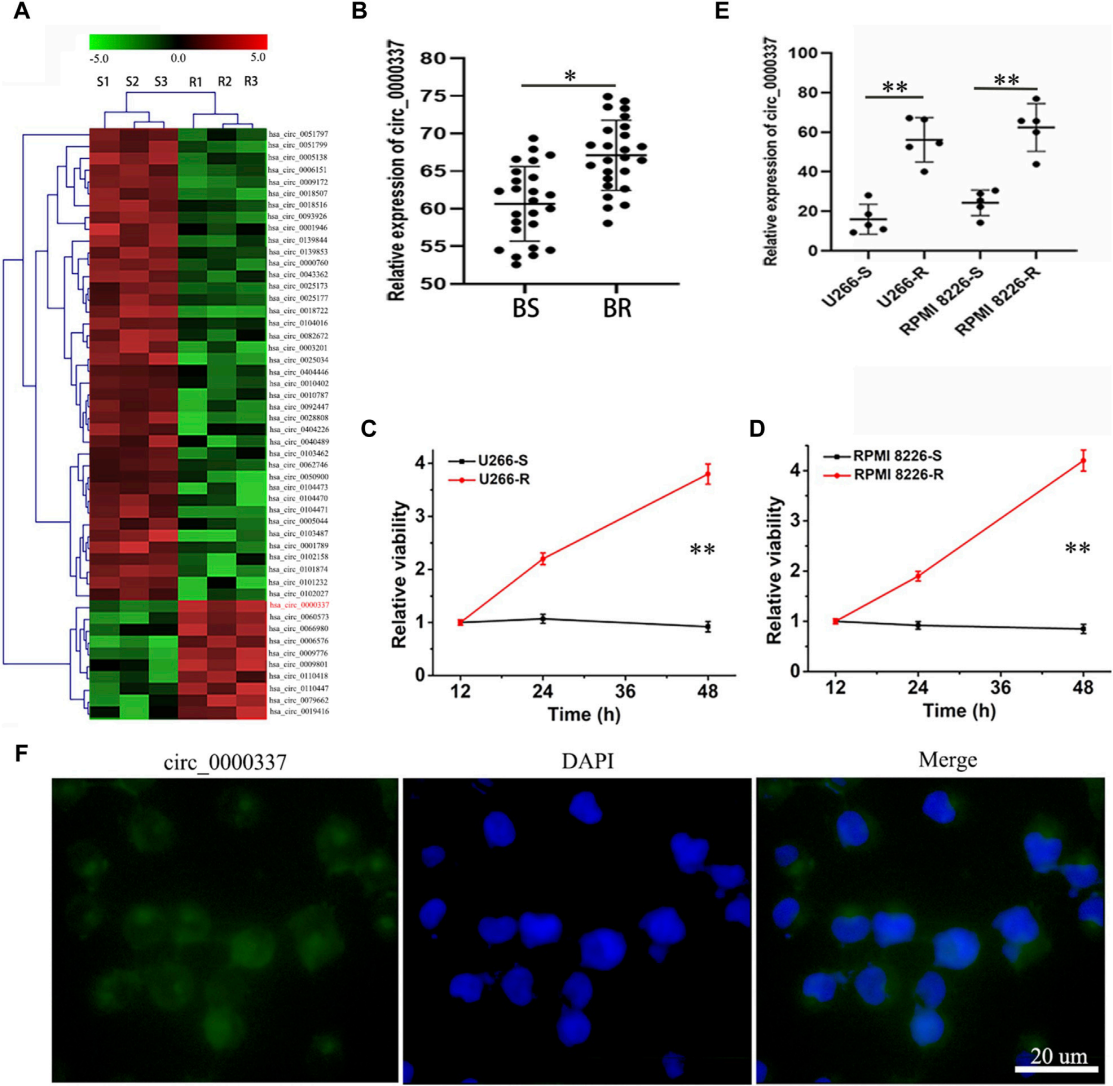
circRNA_0000337 is upregulated in bortezomib-resistant multiple myeloma. **(A)** Heat maps of differentially expressed circRNAs in bortezomib-sensitive and resistant-multiple MM tissues. **(B)** The expression of circRNA_0000337 in the bone marrow of bortezomib-sensitive and bortezomib-resistant MM patients was analyzed by RT-qPCR. Construction and drug resistance detection of bortezomib-resistant MM cell lines U266-R **(C)** and RPMI 8266-R **(D)** (10 nM). **(E)** The expression of circRNA_0000337 in bortezomib-sensitive (U266-S, RPMI 8226-S) and bortezomib-resistant (U266-R, RPMI 8226-R) MM cells was analyzed by RT-qPCR. **(F)** Fluorescence *in situ* hybridization was used to locate circRNA_0000337 (green) in U266-R cells. *p < 0.05, **p < 0.01.

First, the expression of circular RNAs in bortezomib-sensitive and bortezomib-resistant MM patients were examined by high-throughput RNA sequencing. Figure 1A shows that a large number of circRNAs are differently expressed in bortezomib-resistant MM patients, among which circ_0000337 was the most significantly overexpressed in the resistant MM tissues. Furthermore, RT-PCR was employed to measure the expression of circ_0000337 in 25 bortezomib-sensitive and 25 bortezomib-resistant MM tissues. As depicted in Figure 1B, the level of circ_0000337 was significantly higher in bortezomib-resistant MM tissues. To confirm the *in vitro* resistance of MM to bortezomib, we developed 2 MM cell lines (U266-R and RPMI 8226-R) that were resistant to bortezomib. As shown in Figures 1C,D, the sensitivity of U266-R and RPMI 8226-R cells to bortezomib decreased dramatically. Consistent with these findings, circ_0000337 expression was also significantly upregulated in the bortezomib-resistant U266-R and RPMI 8226-R cells (Figure 1E). The function of a circRNA may be influenced by its cellular localization; Figure 1F confirms that circ_0000337 is predominantly localized in the cytoplasm, as determined by fluorescence *in situ* hybridization (FISH). In summary, our research identified circ_0000337 associated with bortezomib-resistant MM, localized in the cytoplasm and significantly upregulated in bortezomib-resistant MM.

### 3.2 circRNA_0000337 is critical for maintaining resistance to bortezomib

**FIGURE 2:**
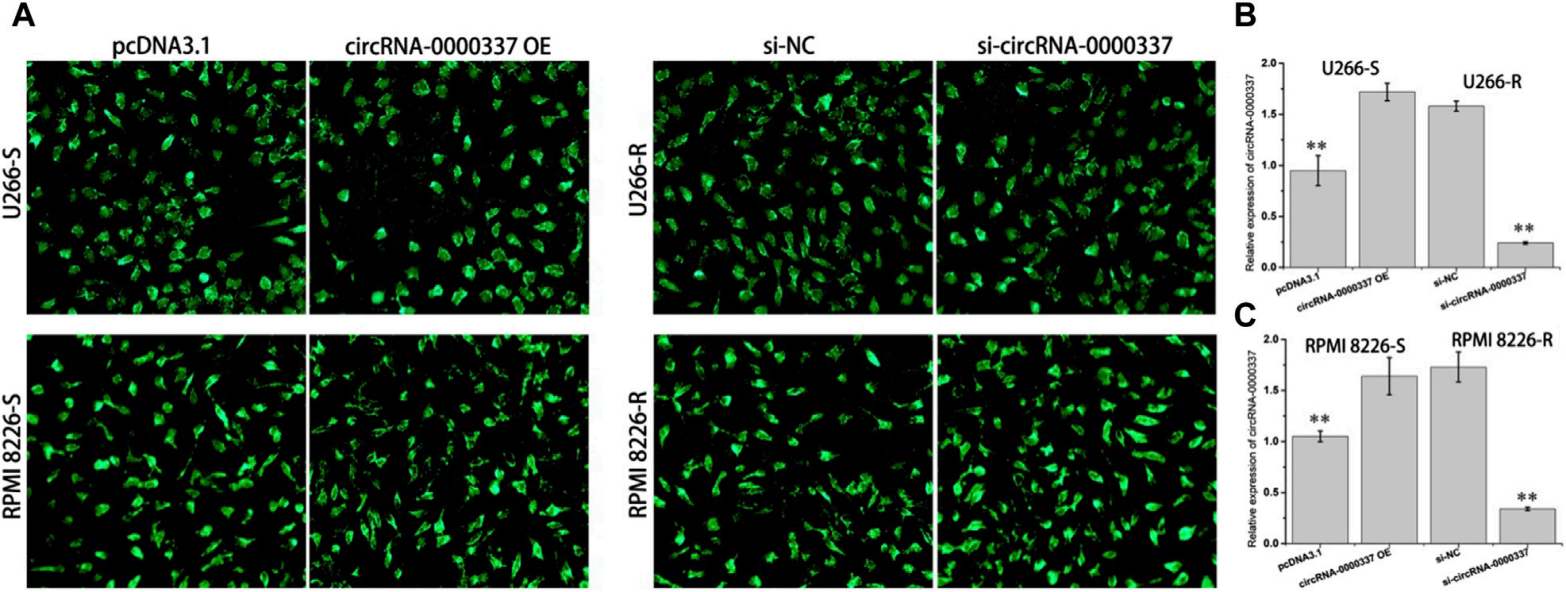
Transfection efficiency of circ_0000337 OE and siRNA vector. **(A)** Fluorescence of GFP after transfection with circ_0000337 OE and siRNA vector. **(B, C)** The expression of circ_0000337 in each group was analyzed by RT-PCR. **p < 0.01.

To explore the role of circ_0000337 in bortezomib resistance, we transfected bortezomib-sensitive U266-S and RPMI 8226-S cells with circ_0000337 high expression plasmid. circRNA_0000337 knockdown was performed on bortezomib-resistant U266-R and RPMI 8226-R cells using siRNA. As shown in Figure 2A, both circ_0000337 OE and siRNA vectors showed high transfection efficiency. Following transfection, circ_0000337 expression in U266-S and RPMI 8226-S cells was significantly upregulated, while circ_0000337 expression in U266-R and RPMI 8226-R cells was significantly downregulated (Figures 2B,C).

**FIGURE 3:**
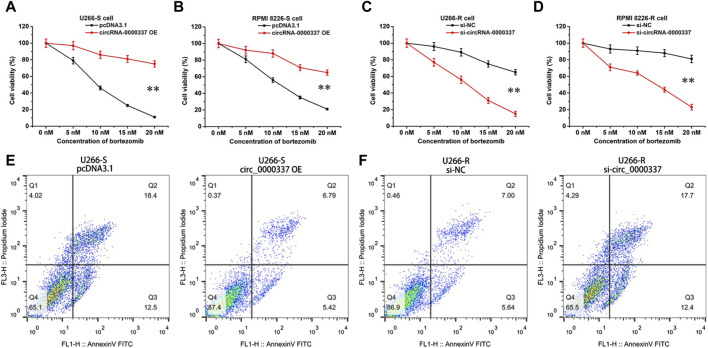
circ_0000337 promotes cell proliferation and drug resistance. **(A, B)** Cell viability of U266-S and RPMI 8226-S cells transfected with circRNA_0000337 after treatment with bortezomib at different concentrations. **(C, D)** Cell viability of U266-R and RPMI 8226-R cells transfected with si-circ_0000337 after treatment with bortezomib. **(E)** The apoptosis of U266-S cells treated with bortezomib (5 nM) before and after transfection with circ_0000337 was detected by flow cytometry. **(F)** The apoptosis of U266-R cells treated with bortezomib (5 nM) before and after transfection with si-circ_0000337 was detected by flow cytometry. **p < 0.01.

In addition, we examined the sensitivity of circ_0000337 to bortezomib in MM cells before and after upregulation and knockdown by CCK8 assay. In U266-S and RPMI 8226-S cells, sensitivity to bortezomib decreased upon upregulation of circ_0000337 (Figures 3A,B). In U266-R and RPMI 8226-R cells, sensitivity to bortezomib was increased when circ_0000337 was knocked down (Figures 3C,D). In U266-S cells, the apoptosis rate induced by bortezomib was significantly reduced when circ_0000337 was upregulated (Figure 3E). In U266-R cells, the apoptosis rate induced by bortezomib was significantly increased when circ_0000337 was downregulated (Figure 3F). These results suggest that circ_0000337 is essential for maintaining resistance to bortezomib, and silencing circ_0000337 significantly improves the efficacy of bortezomib by inducing apoptosis.

### 3.3 circ_0000337 sustains bortezomib resistance by acting as a miRNA sponge for miR-98-5p

**FIGURE 4:**
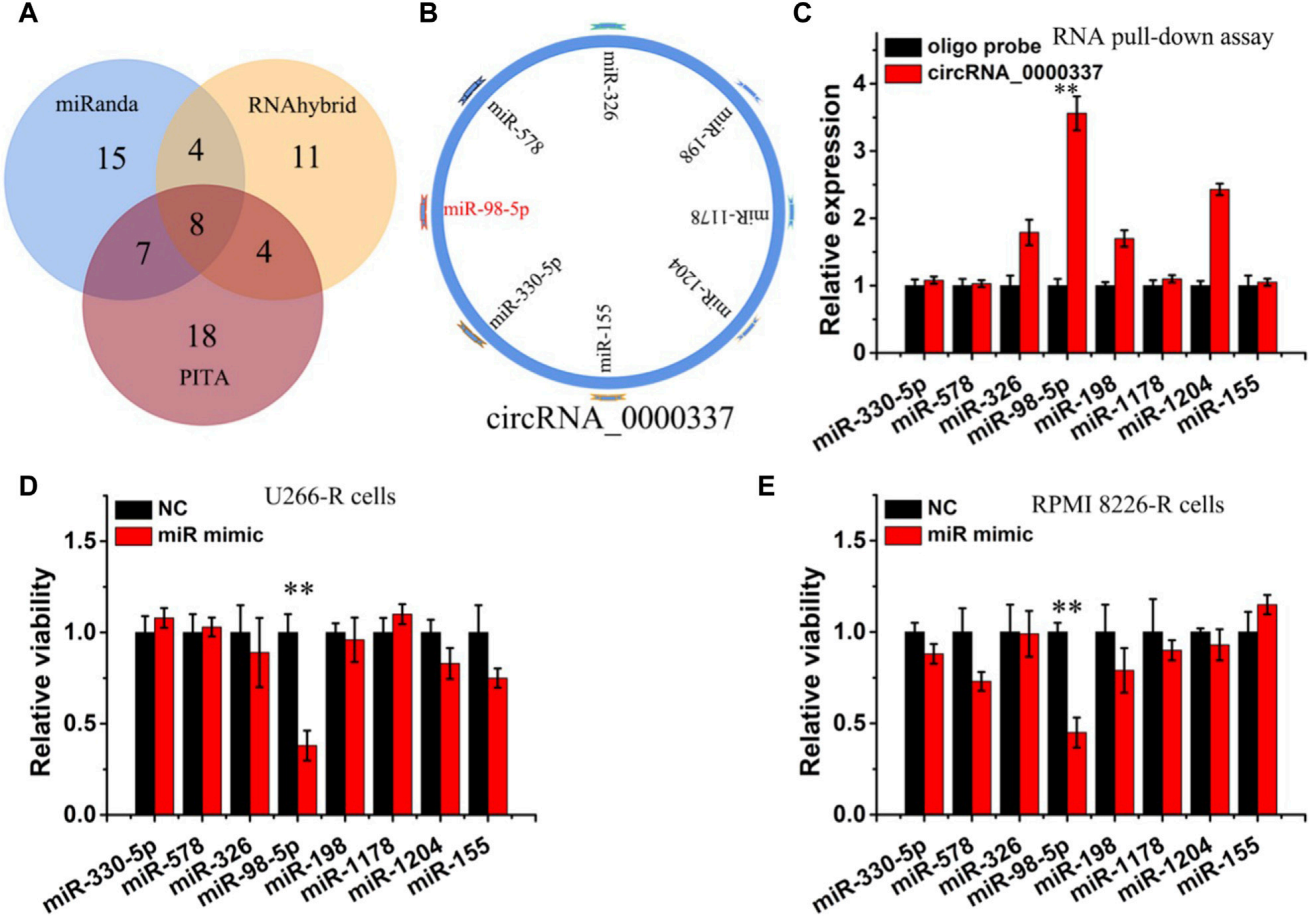
circ_0000337 was regulated by miR-98-5p **(A, B)** The target miRNA of circ_0000337 was inferred by RNAhybrid, miRanda, and PITA. **(C)** RT-qPCR analysis of miRNA level of circ_0000337 binding in pull-down experiment. **(D, E)** Cell viability of U266-R and RPMI 8226-R cells transfected with miRNA mimics was detected by CCK-8 assay. **p < 0.01.

circRNA can act in a variety of ways in the cytoplasm, including sponge miRNA, interacting with proteins, regulating gene transcription, and encoding proteins (Zhou et al., 2023). Since the above result found that circ_0000337 is localized in the cytoplasm, we hypothesize that the miRNA sponge may be a potential mechanism of circ_0000337. miRNA targets interacting with circRNA_0000337 were predicted using miRanda, RNAhybrid, and PITA software (Figures 4A,B). In the predicted miRNAs, miR-330-5p, miR-578, miR-326, miR-98-5p, miR-198, miR-1178, miR-1204, and miR-155 were found to be associated with circ_0000337. The miRNA pull-down assay indicated that miR-98-5p and miR-1204 were obviously enriched in circRNA_0000337 (Figure 4C). After transfecting these miRNA mimics, it was found that miR-98-5p reduced cell viability in U266-R and RPMI 8226-R cells (Figures 4D,E).

**FIGURE 5:**
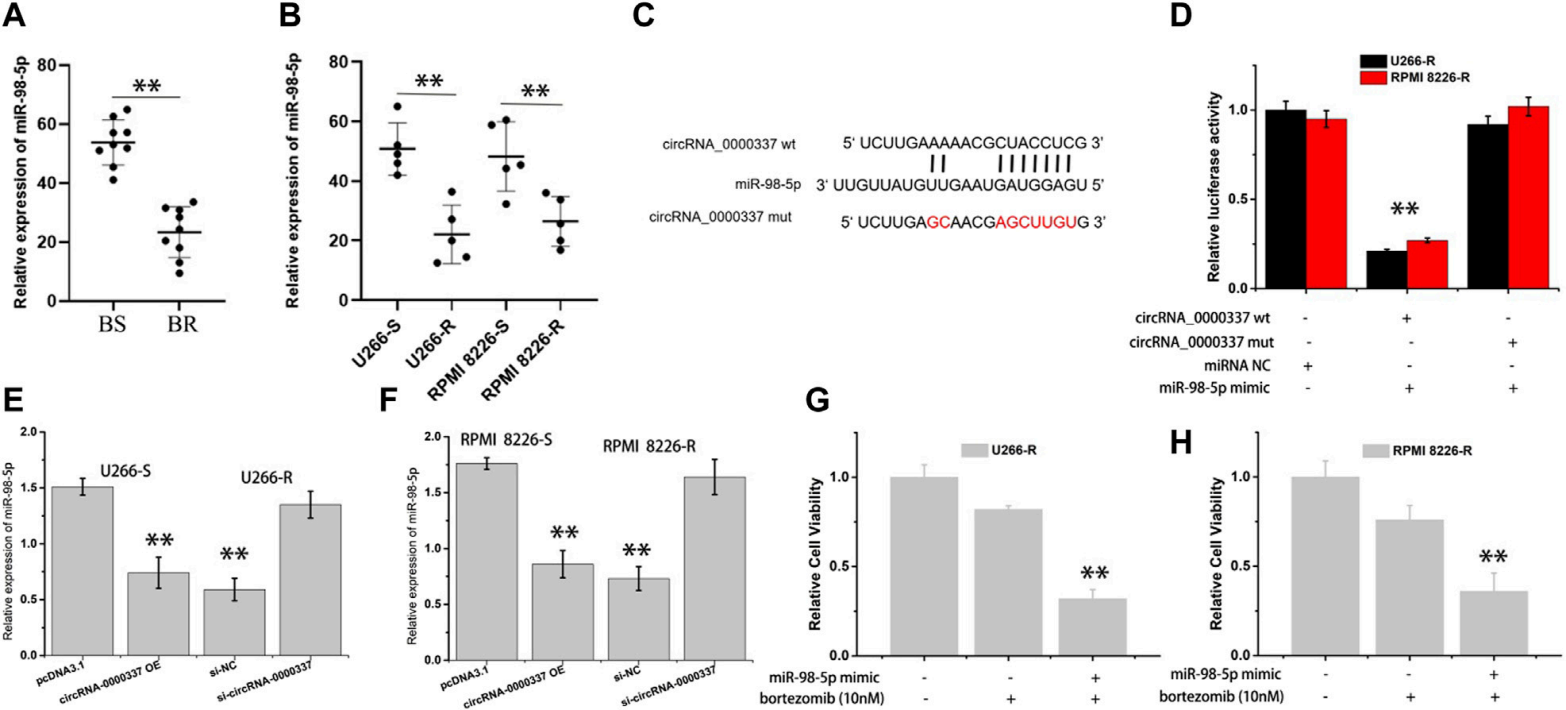
circ_0000337 as the sponge of miR-98-5p. **(A, B)** The expression level of miR-98-5p in bortezomib-sensitive and resistant MM tissues and cells were analyzed by RT-qPCR. **(C)** Schematic diagram of potential binding sites of miR-98-5p to the 3′-UTR of circ_0000337. **(D)** Luciferase reporter assays using the linear form of wild-type and mutant circ_0000337 in U266-R and RPMI 8226-R cells transfected with miR-98-5p mimic. **(E, F)** The expression of miR-98-5p was analyzed by RT-qPCR when circ_0000337 was expressed differently in U266 and RPMI 8226 cells. **(G, H)** The sensitivity of U266-R and RPMI 8226-R cells to bortezomib after transfecting miR-98-5p mimic was detected by CCK-8 assay. **p < 0.01.

In addition, the expression of miR-98-5p in bortezomib-sensitive and resistant MM was analyzed by RT-qPCR. As indicated in Figures 5A,B, miR-98-5p was obviously downregulated in bortezomib-resistant tissues and cells of MM. Based on the miRNA binding site predicted on circ_0000337, luciferase reporter gene detection indicated that miR-98-5p could specifically target wild-type circ_0000337, resulting in decreased luciferase activity (Figures 5C,D). Furthermore, we transfected circ_0000337 high-expression plasmid in U266-S and RPMI 8226-S cells and transfected circ_0000337 knockdown plasmid in U266-R and RPMI 8226-R cells to measure the expression of miR-98-5p. As shown in Figures 5E,F, circ_0000337 was negatively correlated with miR-98-5p. At the same time, we used CCK-8 assays to detect the sensitivity of bortezomib to U266-R and RPMI 8226-R cells transfected with miR-98-5p mimic. In Figures 5G,H, by transfecting mimic of miR-98-5p, it was found that miR-98-5p reduced bortezomib resistance in U266-R and RPMI 8226-R cells. This suggests that circ_0000337 has a miRNA sponge effect on miR-98-5p and maintains bortezomib resistance in MM.

### 3.4 DNA2 is a regulatory target of miR-98-5p in bortezomib-resistant MM

**FIGURE 6:**
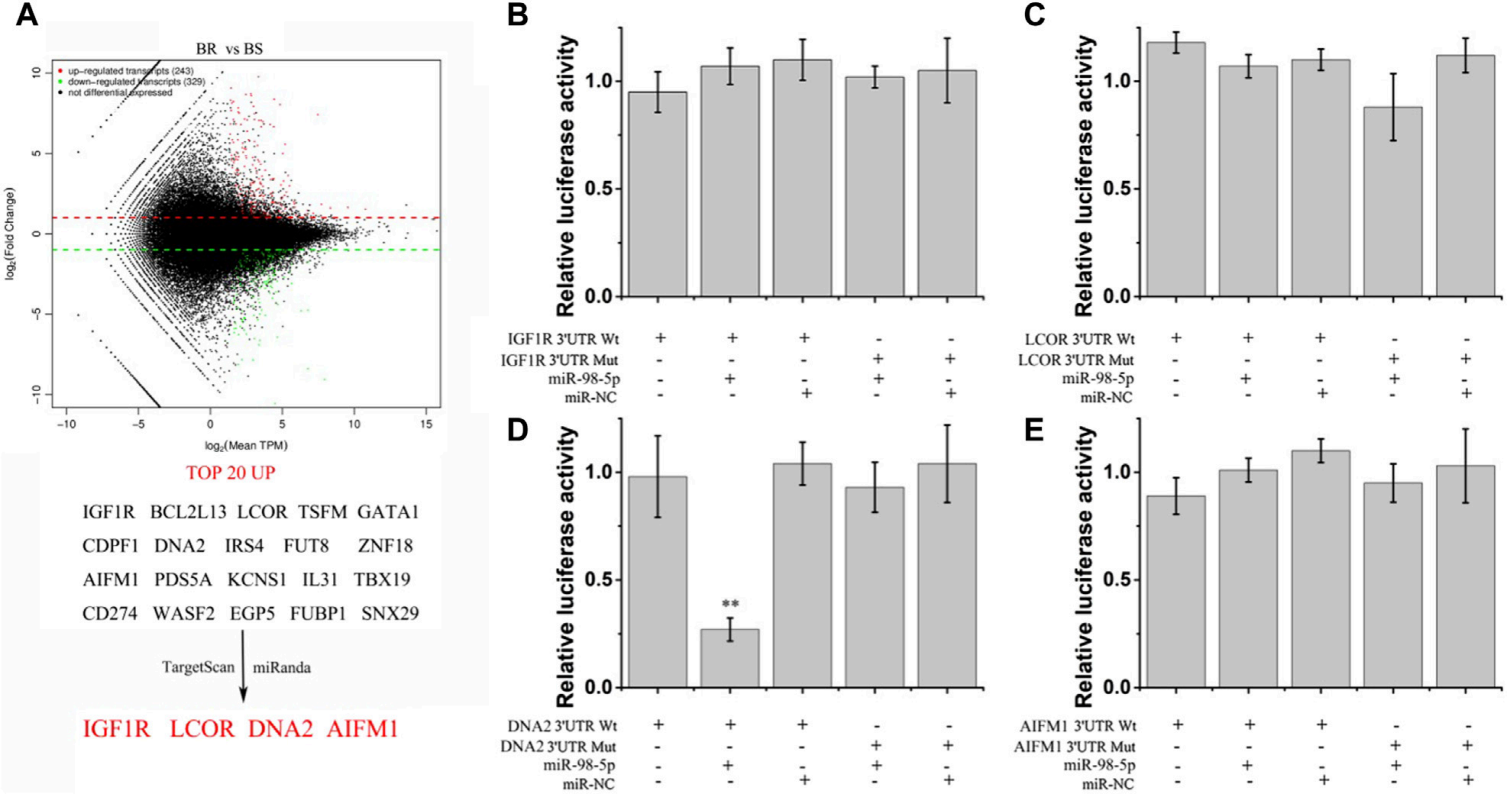
Screening of miR-98-5p target genes. **(A)** Differently-expressed genes in bortezomib-resistant and sensitive MM tissues and potential target genes of miR-98-5p screened by miRanda and TargetScan software. **(B–E)** The relative luciferase activity of wild-type and mutant IGF1R, LCOR, DNA2, AIFM1, and miR-98-5p co-transfection in U266 cells. **p < 0.01.

Differentially expressed mRNA of bortezomib sensitive and resistant tissues was identified by high-throughput sequencing (Figure 6A). The first 20 upregulated mRNAs were then predicted by miRanda and TargetScan. We found the 3′UTR of IGF1R, LCOR, DNA2, and AIMF1 may be the target of miR-98-5p. Whether miR-98-5p can directly target IGF1R, LCOR, DNA2, and AIMF1 in U266 cells was determined by luciferase reporter gene assay (Figures 6B–E). In U266 cells transfected with miR-98-5p, the luciferase activity of a reporter with wild-type miR-98-5p sites of DNA2 was significantly decreased compared with that with mutated sites (Figure 6D). Therefore, miR-98-5p can directly target DNA2.

**FIGURE 7:**
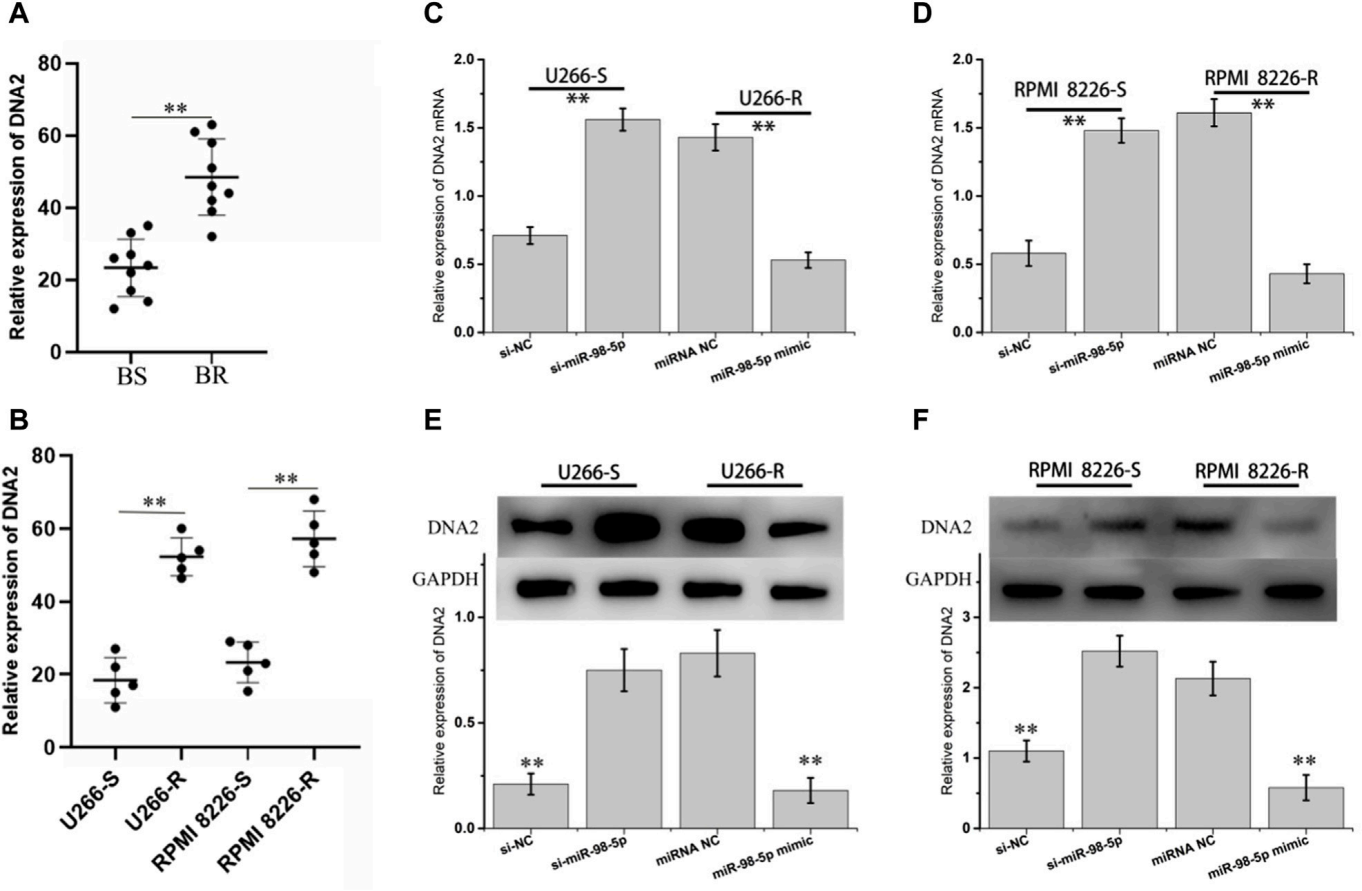
DNA2 is the direct target of miR-98-5p. **(A, B)** The mRNA expression of DNA2 in bortezomib sensitive and resistant MM tissues and cells. **(C, D)** The mRNA expression of DNA2 at different expressions of miR-98-5p was analyzed by RT-qPCR. **(E, F)** The protein expression levels of DNA2 in different expressions of miR-98-5p were detected by Western blot. **p < 0.01.

The DNA replication helicase/nuclease 2 (DNA2) gene encodes a member of the helicase family (Wang et al., 2021). The encoded protein is a conserved helicase/nuclease involved in maintaining the stability of mitochondrial and nuclear DNA (Sun et al., 2022a). It was found that overexpressed DNA2 promotes tumor cell survival by overcoming replication stress on DNA replication forks induced by chemotherapy or radiotherapy (Kumar et al., 2017; Han et al., 2021). First, we detected the expression of DNA2 mRNA in bortezomib-sensitive and drug-resistant tissue and cells, as indicated in Figures 7A,B. DNA2 mRNA was obviously upregulated in bortezomib-resistant tissue and cells. Secondly, we performed knockdown and high expression treatment of miR-98-5p in MM cells. In Figures 7C–F, after miR-98-5p was knocked down in U266-S and RPMI 8226-S, the expression of DNA2 mRNA and protein was obviously upregulated. When miR-98-5p was highly expressed in U266-R and RPMI 8226-R, DNA2 mRNA and protein were obviously downregulated. These results indicate that circ_0000337 can specifically sponge miR-98-5p as ceRNA, activate DNA2-mediated DNA repair, and promote bortezomib resistance.

### 3.5 The stability of circ_0000337 is modulated by m^6^A RNA methylation

**FIGURE 8:**
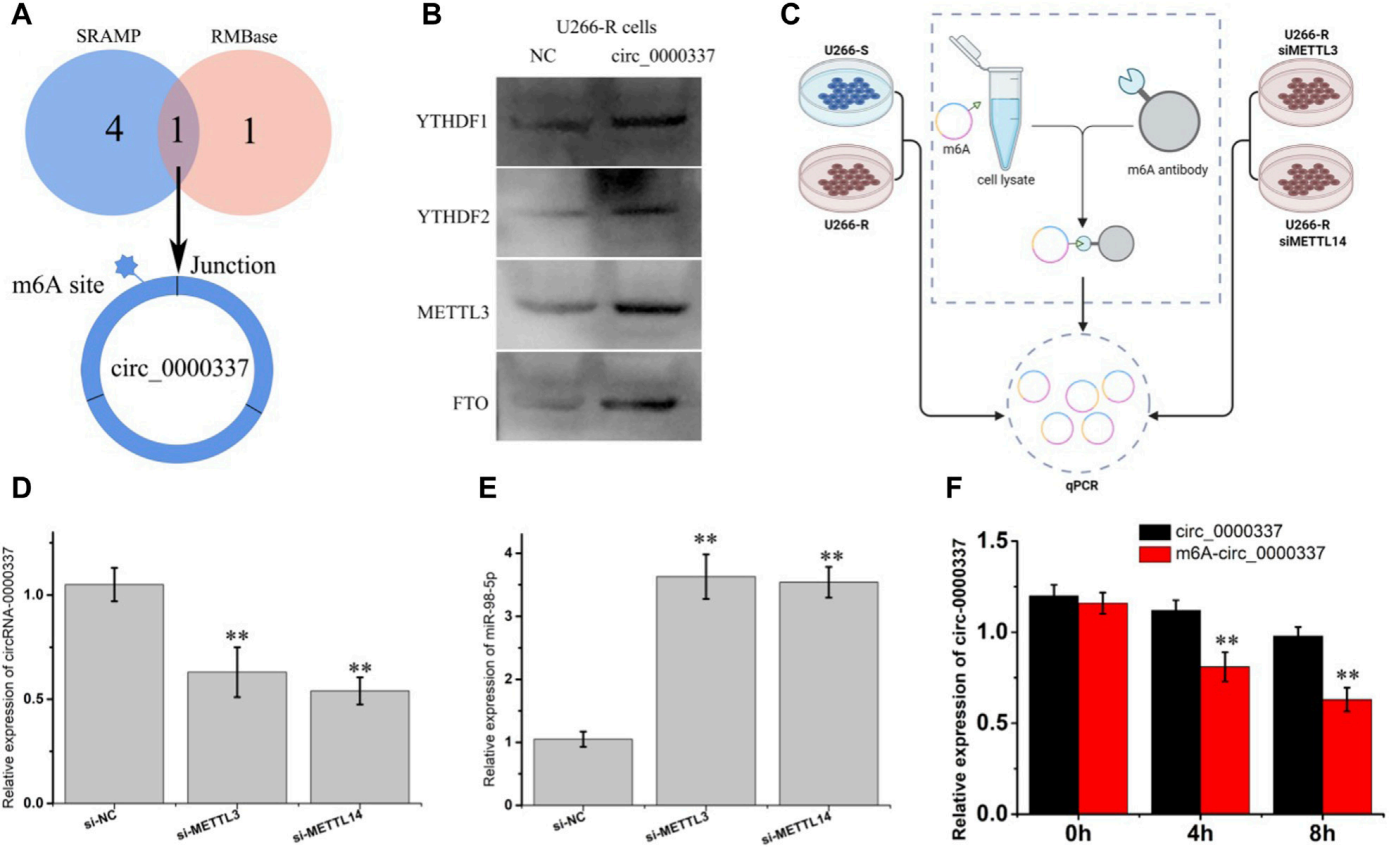
circ_0000337 is regulated by m6A methylation. **(A)** The m^6^A site in circ_0000337 was predicted by SRAMP and RMBase v2.0 software. **(B)** YTHDF1/2, METTL3 and FTO were detected by western blot after pulldown using the circ_0000337 probe. **(C)** Flow chart of m^6^A-circ_0000337 detection using a m^6^A antibody. **(D)** The expression of circ_0000337 was measured by PCR after transfection with si-METTL3 and si-METTL14 into U266-R cells. **(E)** The expression of miR-98-5p was measured by PCR after transfection with si-METTL3 and si-METTL3 in U266-R cells. **(F)** The expression of circ_0000337 was measured by PCR in U266 cells over-expressing circ_0000337 with or without the m^6^A site mutated under actinomycin D (5 μg/mL) treatment for 0, 4, 8 h **p < 0.01.

circ_0000337 may be produced by pre-mRNA back-splicing of exons 17, 18, and 19 of PPFIA1. However, the detailed mechanism controlling hsa_circ_0000337 levels remain unclear. m^6^A is one of the most abundant base modifications in RNA and may regulate circRNA biological functions (Li et al., 2023a). SRAMP and RMBase v2.0 software were used to predict the m^6^A location in circRNA_0000337, we found an m^6^A site near the junction region (Figure 8A). During m^6^A methylation, m^6^A methyltransferases (METTL3 and METTL14) and m^6^A demethylases (FTO and ALKBH5) together determine how much m^6^A is distributed on RNA. Readers (such as YTHDF1 and YTHDF2) mainly recognize the m^6^a site and mediate the corresponding function (Zhang et al., 2021a). As shown in Figure 8B, the pull-down experiment showed that circRNA_0000337 acted with m^6^A-related proteins (METTL3, FTO, and YTHDF1/2) in bortezomib-resistant cells. Furthermore, the expression level of m^6^A-circRNA_0000337 in cells was detected by qPCR after capturing m^6^A-circRNA_0000337 using m^6^A antibody (Figure 8C). As shown in Figures 8D,E, the expression of m^6^A-circRNA_0000337 was the highest in U266-R cells. However, after knocking down METTL3 and METTL14 in U266-R cells, the expression of m^6^A-circRNA_0000337 was significantly decreased, while the expression of miR-98-5p was increased. RNA stability tests using actinomycin D showed that wild-type circ_0000337 had higher stability than m^6^A-mutated circ_0000337 (Figure 8F). The above results indicated that circ_0000337 was adjusted by m^6^A modification in the process of bortezomib resistance, and m^6^A modification regulates the expression of circ_0000337 by increasing its stability.

### 3.6 *In vivo* targeting circ_0000337 reverses MM bortezomib resistance

**FIGURE 9:**
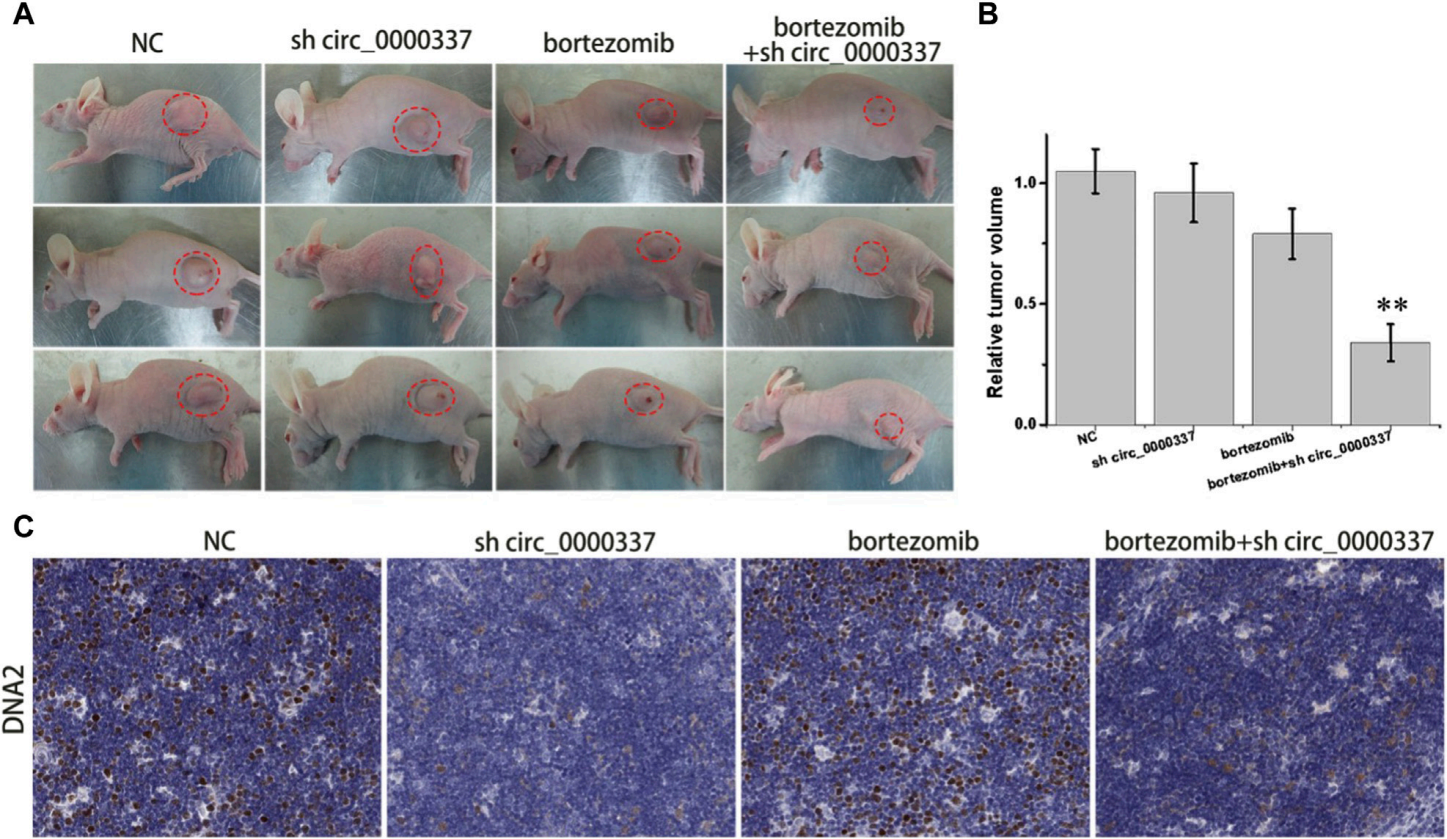
*In vivo* targeting circ_0000337 reverses MM bortezomib resistance. **(A)** Tumor images of nude mice in different treatment groups. A tumor-bearing mouse model was constructed by subcutaneous injection of U266-R cells. The lentivirus shcirc_0000337 was injected locally around the tumor and treated with bortezomib (0.5 mg/kg, intraperitoneal injection) for 2 weeks. **(B)** The relative tumor volume in nude mice in different treatment groups. **(C)** DNA2 immunohistochemical staining (brown) of tumor tissue in different treatment groups. **p < 0.01.

To further validate the results of the *in vitro* study, a tumor-bearing mouse model was constructed by subcutaneously injecting bortezomib-resistant U266-R cells. As indicated in Figures 9A,B, compared with the NC group, the tumor volume in the bortezomib group only decreased slightly, while the tumor volume in the bortezomib + sh circ_0000337 group was significantly reduced. Similar to the results of *in vitro* experiments, immunohistochemistry staining indicated a significant decrease of DNA2 in the shcirc_0000337 group (Figure 9C). The above results confirm that targeted intervention of circ_0000337 to regulate the expression of DNA2 can reverse bortezomib resistance in MM patients.

## 4 Discussion

Multiple myeloma (MM) is the most common type of malignant plasmacytosis (Robinson et al., 2023). Due to slow onset and no obvious symptoms in the early stage, MM is easy to be misdiagnosed (Abeykoon et al., 2021). The current treatment methods mainly include symptomatic treatment, chemotherapy, CAR-T cell therapy, and hematopoietic stem cell transplantation (Iskrzak et al., 2022). Bortezomib is currently one of the first-line drugs for multiple myeloma (Roussel et al., 2022). Bortezomib can directly bind to the active site of the 20S core, inhibit the activity of 26S proteasome, cause IKB aggregation, prevent the release of NF-KB, and thereby inhibit tumor growth (Federico et al., 2020). However, with the application of bortezomib, the problem of drug resistance has gradually emerged (Bao et al., 2023). The mechanisms of resistance may primarily involve the overexpression of multidrug-resistant proteins such as P-glycoprotein in cells, decreased binding affinity of bortezomib due to intracellular genetic alterations, and abnormalities in the ubiquitination pathway (Li et al., 2022a; Wang et al., 2022; Wang et al., 2023). In this study, we have established bortezomib-resistant cell lines and a mouse model to simulate bortezomib resistance in MM patients.

circRNA is a class of ncRNAs without a 5′-terminal cap and a 3′-terminal poly(A) tail (Dong et al., 2023). circRNA has a closed ring structure with covalent bonds and is not easily degraded by exonuclease RNaseR (Zhang et al., 2021c). It is more stable than linear RNA and is involved in many diseases and tumor processes (Li et al., 2022b). Numerous studies have shown that circRNA functions by acting as ceRNA, regulating transcription splicing and expression, and interacting with RNA-binding proteins (Sun et al., 2022b). circRNA generally contains multiple miRNA binding sites, and research has found that hsa_circ_0134426 was down-expressed in MM. Hsa_circ_0134426 can upregulate NDNF expression by inhibiting miR-146b-3p, thus blocking MM cell growth, colony formation, and migration (Li et al., 2023b). In MM patients and cell lines, the over-expressed CircATIC can accelerate the proliferation, migration, invasion, and glycolysis of MM cells by mediating the miR-324-5p/HGF signaling pathway and inhibiting the apoptosis of MM cells (Wu et al., 2022). However, the relationship between circRNA and bortezomib resistance in MM is not clear. Therefore, this study investigated the expression of circRNA in bortezomib-resistant patients by high-throughput sequencing. In this study, circ_0000337 was significantly upregulated in bortezomib-resistant cells and tumor-bearing mouse models. Silencing circRNA_0000337 could effectively reverse bortezomib resistance. In particular, we found that circRNA_0000337 can specifically bind miR-98-5p as a miRNA sponge, upregulate DNA2 expression, and induce bortezomib resistance.

m^6^A is one of the most abundant RNA modifications in eukaryotes (Chen et al., 2023). The methylation of adenine is catalyzed by a methyltransferase complex (METTL3\METTL14), and demethylation can be catalyzed by FTO and AKLBH5 (Zhang et al., 2021b). Studies have found that isocitrate dehydrogenase 2 (IDH2) is highly expressed in MM cells, which can regulate the methylation of WNT7B and activate the Wnt signaling pathway by activating RNA demethylase FTO, thus promoting the tumorigenesis and progression of MM (Song et al., 2021). Studies have found that METTL3 can upregulate the expression of BZW2 by regulating m6A modification and promote the progress of MM by inhibiting apoptosis (Huang et al., 2023). However, the mechanism of m^6^A-modified circRNA in bortezomib-resistant MM is unclear.

In this research, SRAMP and RMBase v2.0 software were used to predict the m^6^A site of circ_0000337. It was found that in bortezomib-resistant tissues and cells, the level of m^6^A in circ_0000337 increased, and when the m^6^A modification was inhibited, the expression of circ_0000337 decreased. The preliminary experimental results show that m^6^A modification can improve the stability of circ_0000337. The mechanism of increased m^6^A level of circ_0000337 in bortezomib-resistant MM needs further study.

## 5 Conclusion

In summary, the upregulation of circ_0000337 is crucial for maintaining the resistance of bortezomib in MM. Knockdown of circ_0000337 significantly improves the sensitivity of resistant cells to bortezomib. Our findings reveal that circ_0000337 inhibited the expression of miR-98-5p by acting as a miRNA sponge, upregulated the expression of DNA2, regulated the DNA repair process, and induced bortezomib resistance. Furthermore, in bortezomib-resistant cells, an increase in m6A methylation at specific sites of circ_0000337 was observed, which enhanced the stability of circ_0000337. In cell and tumor-bearing mouse models, the knockdown of circ_0000337 with shRNA lentivirus significantly improved the efficacy of bortezomib. This study proposes a new mechanism of bortezomib resistance in MM, suggesting that targeted knockdown of circ_0000337 combined with bortezomib could serve as a novel therapeutic approach for MM.

## Data Availability

The original contributions presented in the study are included in the article/Supplementary Material, further inquiries can be directed to the corresponding authors.
