# Supplementary material for: N6-methyladenosine-modified circ_0000337 sustains bortezomib resistance in multiple myeloma by regulating DNA repair
Source: Front Cell Dev Biol. 2024 Mar 22;12:1383232. doi: 10.3389/fcell.2024.1383232 (PMC10995360; doi:10.3389/fcell.2024.1383232)
Supplement: Supplementary file 1 [file DataSheet1.docx]

Supplementary Material

# Supplementary Figures and Tables

## Supplementary Tables

**Table 1. Primers and RNA sequences used in this study**

| **Primer sequence** |  |  |
| --- | --- | --- |
| hsa_circ_0000337 | Forward | GATGCCTTGGGACTTAGCAA |
|  | Reverse | CGGGGAGGTTTCACACTTTA |
| GAPDH | Forward | AATGGGCAGCCGTTAGGAAA |
|  | Reverse | GCGCCCAATACGACCAAATC |
| DNA2 | Forward | TTTGCCACTGCCTACCAGAG |
|  | Reverse | GAGATACTGGCAGGTCAGGC |
| hsa-miR-330-5p | Forward | AACAAGTCTCTGGGCCTGTG |
| hsa-miR-578 | Forward | AACCGGCTTCTTGTGCTCT |
| hsa-miR-326 | Forward | ttctccaaaagaaagcactttctg |
| hsa-miR-98-5p | Forward | AAGCGACCTGAGGTAGTAAGTT |
| hsa-miR-198 | Forward | AACAAGGGTCCAGAGGGGA |
| hsa-miR-1178 | Forward | AACACGCTTGCTCACTGTTC |
| hsa-miR-1204 | Forward | AACAATTCGTGGCCTGGTC |
| hsa-miR-155 | Forward | AACACGCTTAATGCTAATCGTGA |

## Supplementary Figures


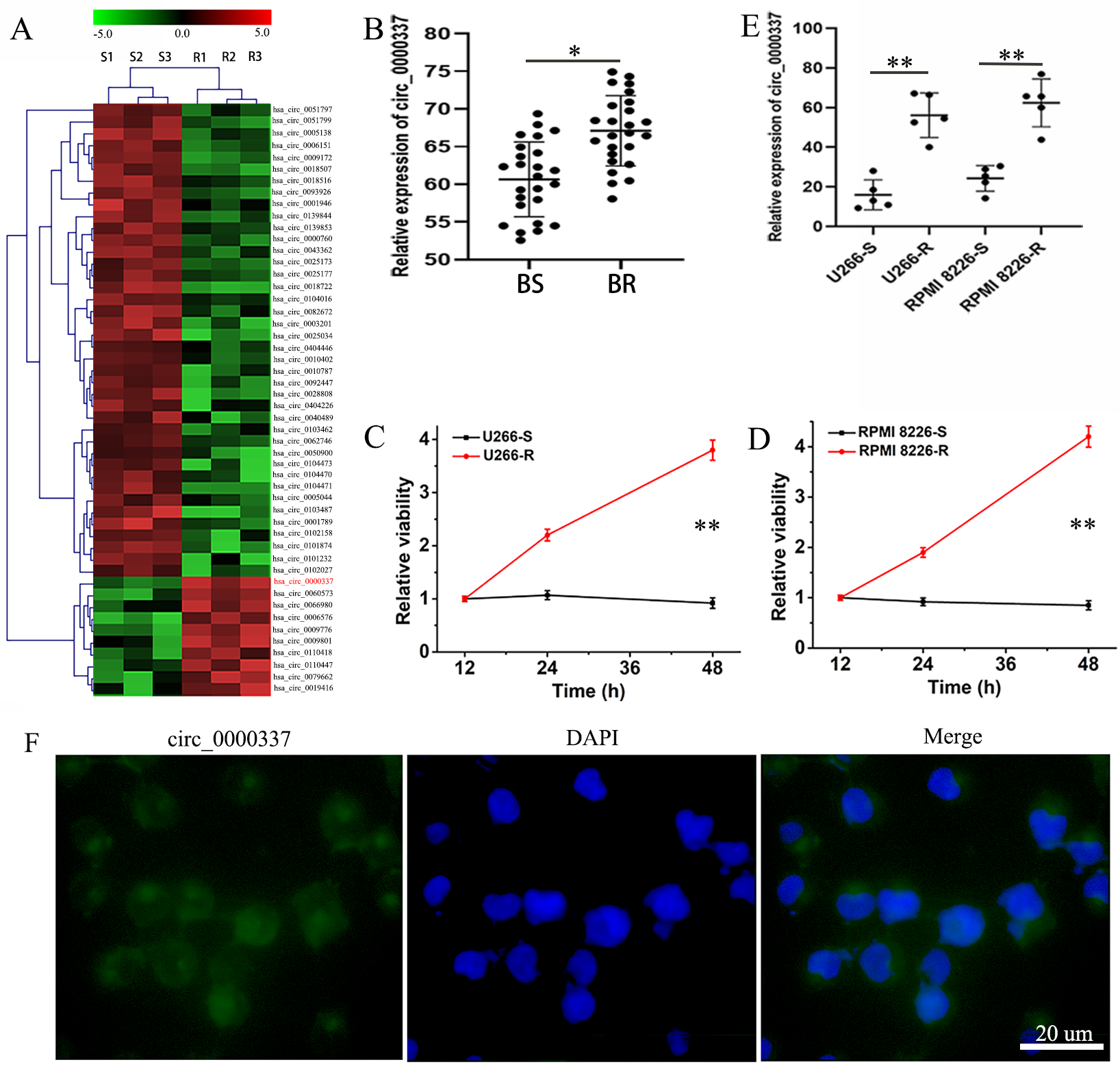


**Figure 1** **circRNA_0000337 is up-regulated in bortezomib-resistance multiple myeloma.** (**A**) Heat maps of differentially expressed circRNAs in bortezomib-sensitive and resistant-multiple MM tissues. (**B**) The expression of circRNA_0000337 in bone marrow of bortezomib-sensitive and bortezomib-resistant MM patients was analyzed by RT-qPCR. Construction and drug resistance detection of bortezomib-resistant MM cell lines U266-R (**C**) and RPMI 8266-R (**D**) (10 nM). (**E**) The expression of circRNA_0000337 in bortezomid-sensitive (U266-S, RPMI 8226-S) and bortezomib-resistant (U266-R, RPMI 8226-R) MMa cells was analyzed by RT-qPCR. (F) Fluorescence in situ hybridization was used to locate circRNA_0000337 (green) in U266-R cells. *p<0.05, **p<0.01.


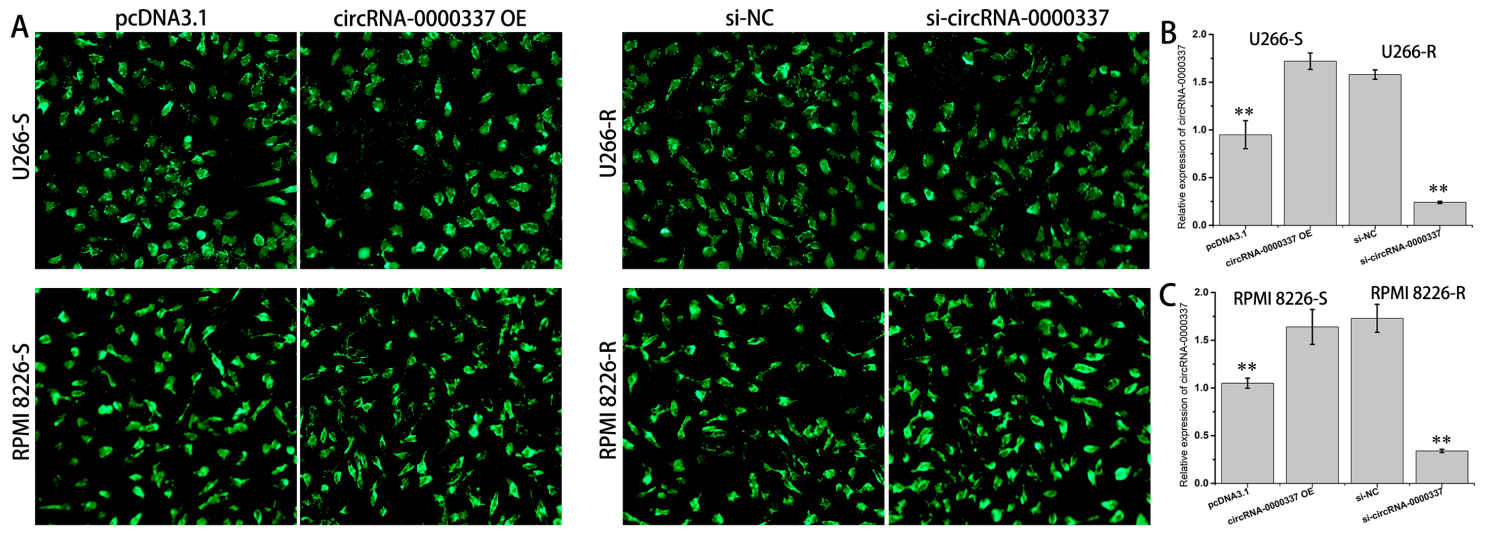


**Figure 2** **Transfection efficiency of circ_0000337 OE and siRNA vector.** (**A**) Fluorescence of GFP after transfection with circ_0000337 OE and siRNA vector. (**B**) The expression of circ_0000337 in each group was analyzed by RT-PCR. **p<0.01.


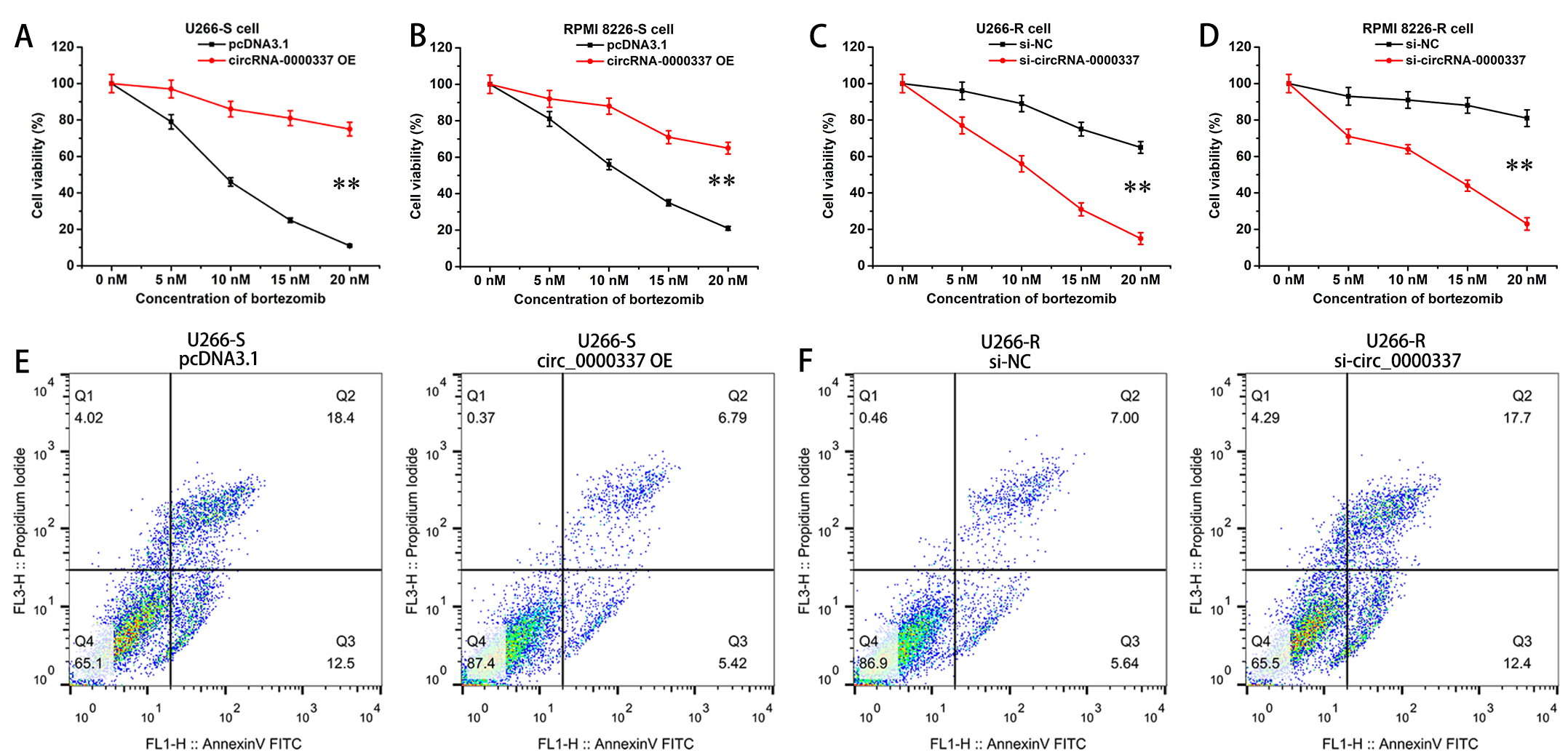


**Figure 3. circ_0000337 promotes cell proliferation and drug resistance.** (**A, B**) Cell viability of U266-S and RPMI 8226-S cells transfected with circRNA_0000337 after treatment with bortezomib at different concentrations. (**C, D**) Cell viability of U266-R and RPMI 8226-R cells transfected with si-circ_0000337 after treatment with bortezomib. (**E**) The apoptosis of U266-S cells treated with bortezomib (5 nM) before and after transfection with circ_0000337 was detected by flow cytometry. (**F**) The apoptosis of U266-R cells treated with bortezomib (5 nM) before and after transfection with si-circ_0000337 was detected by flow cytometry. **p<0.01.


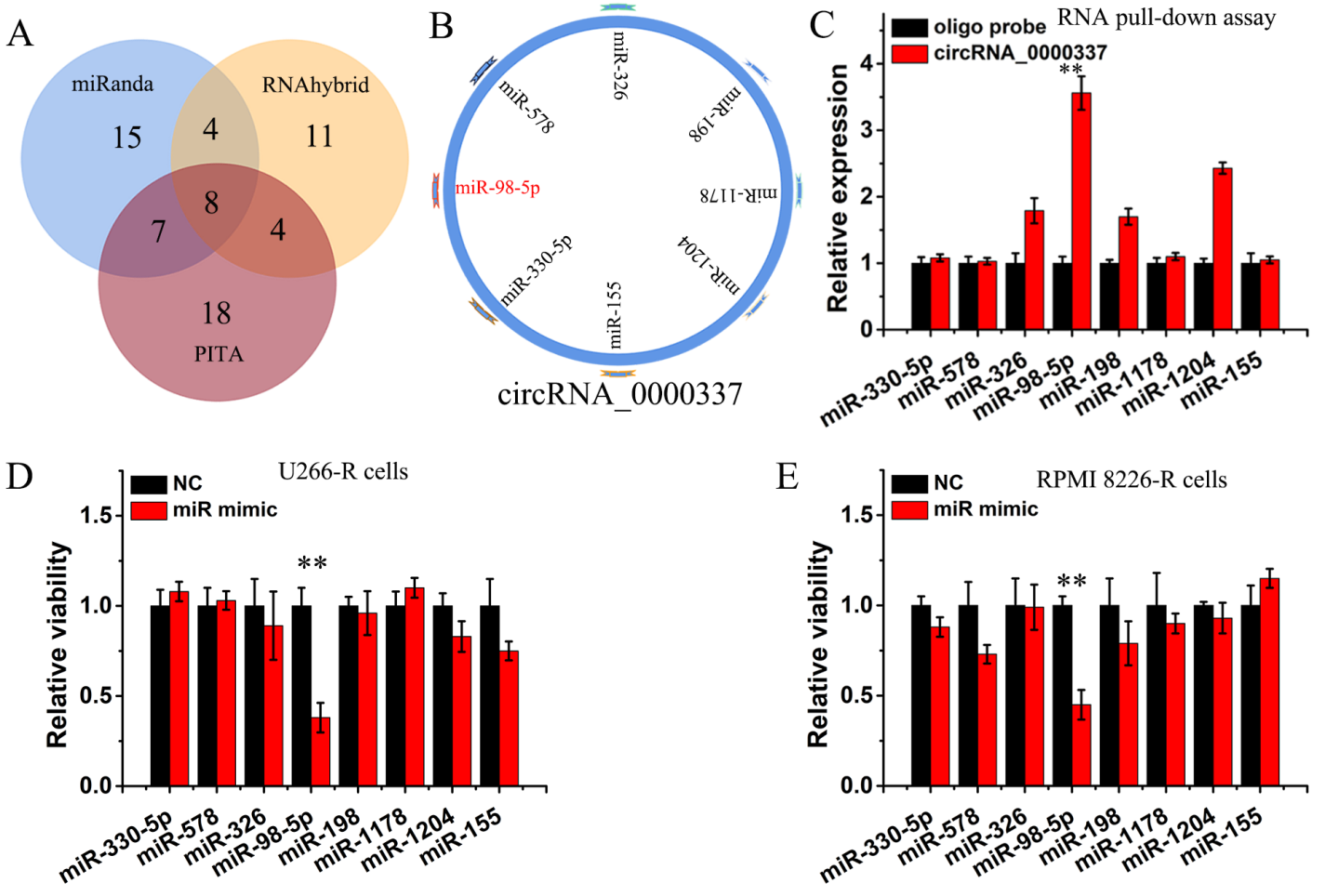


**Figure 4** **circ_0000337 was regulated by miR-98-5p** (**A, B**) The target miRNA of circ_0000337 was inferred by RNAhybrid, miRanda and PITA. (**C**) RT-qPCR analysis of miRNA level of circ_0000337 binding in pull-down experiment. (**D, E**) Cell viability of U266-R and RPMI 8226-R cells transfected with miRNA mimics was detected by CCK-8 assay. **p<0.01.


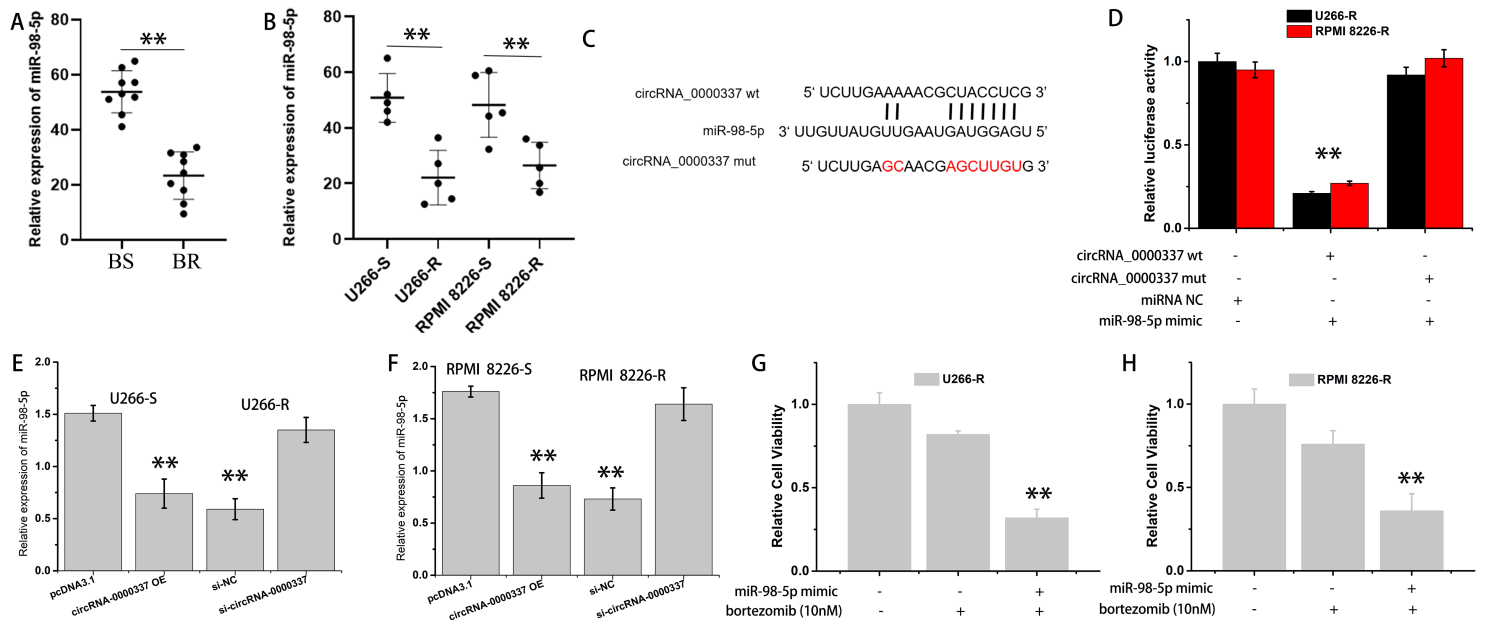


**Figure 5. circ_0000337 as the sponge of miR-98-5p.** (**A, B**) The expression level of miR-98-5p in bortezomib sensitive and resistant MM tissues and cells was analyzed by RT-qPCR. (**C**) Schematic diagram of potential binding sites of miR-98-5p to the 3 '-UTR of circ_0000337. (D)Luciferase reporter assays using the linear form of wild-type and mutant circ_0000337 in U266-R and RPMI 8226-R cells transfected with miR-98-5p mimic. (**E, F**) The expression of miR-98-5p was analyzed by RT-qPCR when circ_0000337 was expressed differently in U266 and RPMI 8226 cells. (**G, H**) The sensitivity of U266-R and RPMI 8226-R cells to bortezomib after transfecting miR-98-5p mimic was detected by CCK-8 assay. **p<0.01.


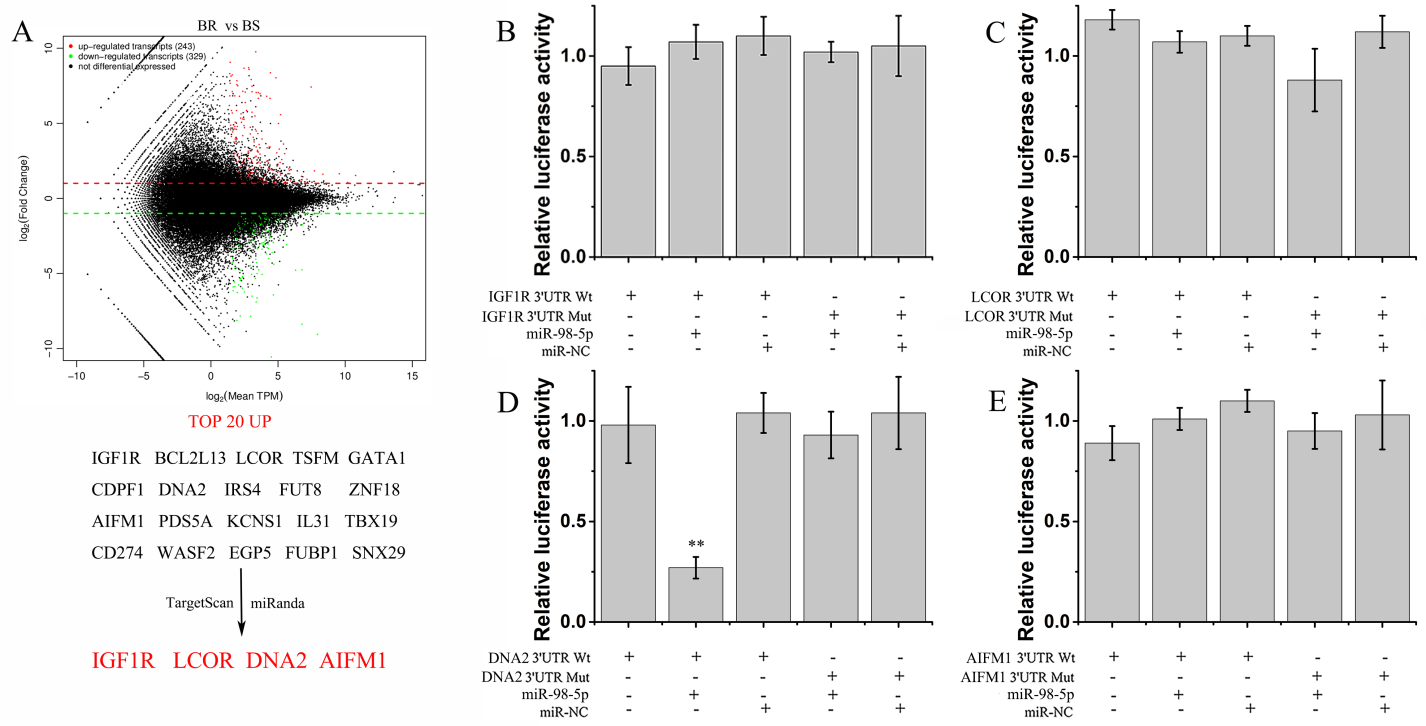


**Figure 6. Screening of miR-98-5p target genes.** (**A**) Differently-expressed genes in bortezomib resistant and sensitive MM tissues and potential target genes of miR-98-5p screened by miRanda and TargetScan software. (**B-E)** The relative luciferase activity of wild-type and mutant IGF1R, LCOR, DNA2, AIFM1 and miR-98-5p co-transfection in U266 cells. **p<0.01.


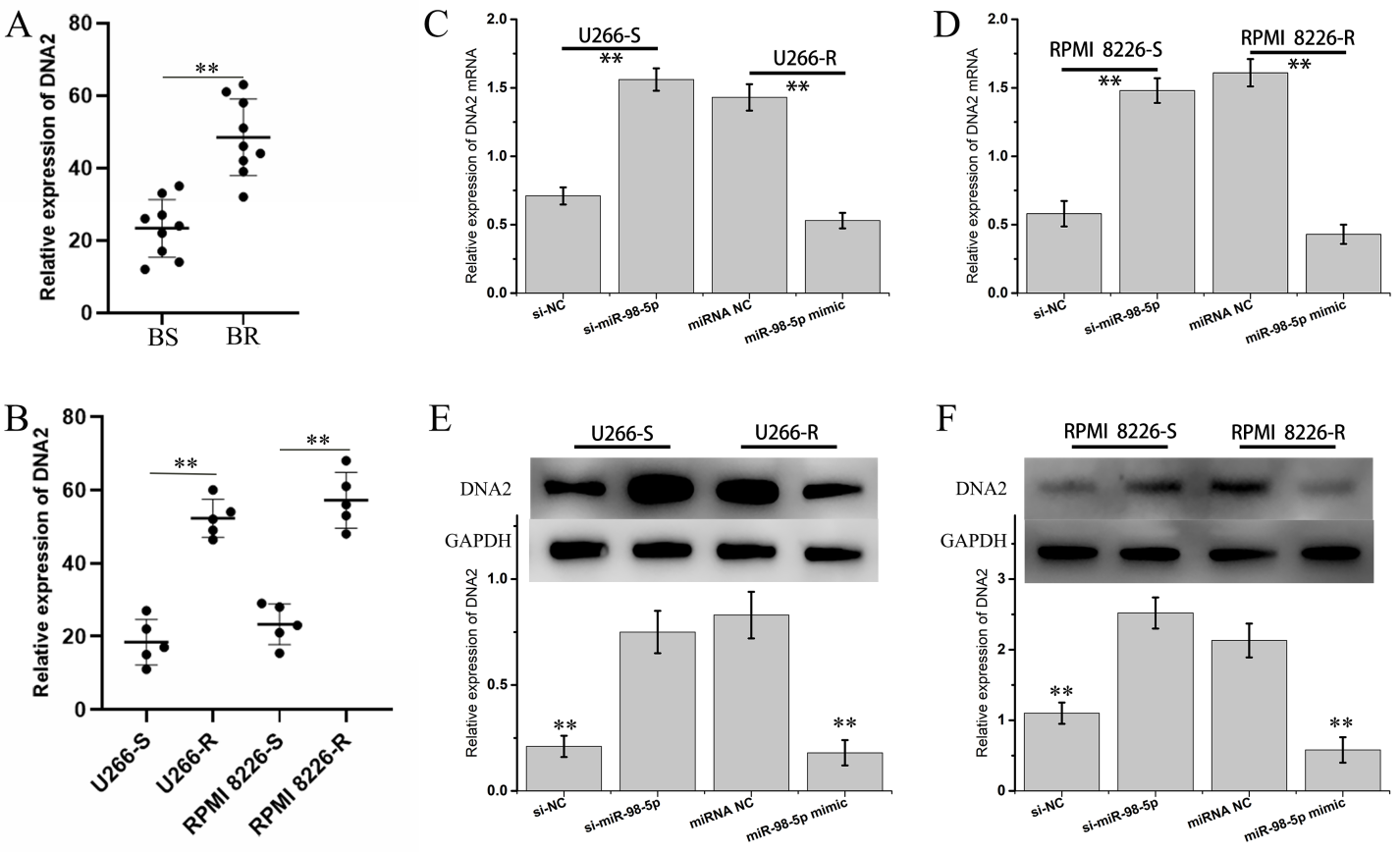


**Figure 7. DNA2 is the direct target of miR-98-5p.** (**A, B**) The mRNA expression of DNA2 in bortezomib sensitive and resistant MM tissues and cells. (**C, D**) The mRNA expression of DNA2 at different expressions of miR-98-5p was analyzed by RT-qPCR. (**E, F**) The protein expression levels of DNA2 in different expressions of miR-98-5p was detected by Western blot. **p<0.01.


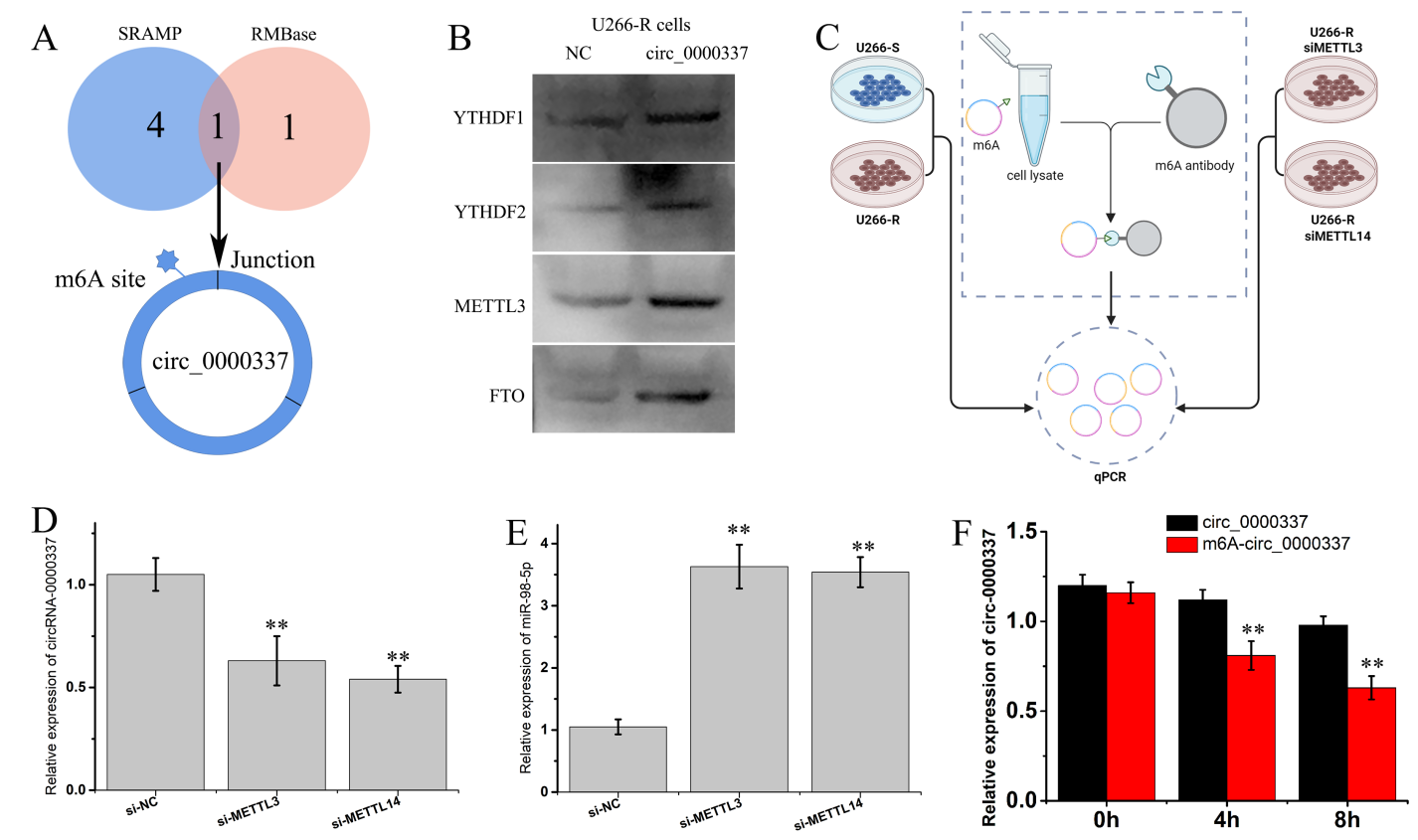


**Figure 8. circ_0000337 is regulated by m^6^A methylation.** (**A**) The m^6^A site in circ_0000337 was predicted by SRAMP and RMBase v2.0 software. (**B**) YTHDF1/2, METTL3 and FTO were detectd by western blot after pulldown using the circ_0000337 probe. (**C**) Flow chart of m^6^A-circ_0000337 detection using a m^6^A antibody. (**D**) The expression of circ_0000337 was measured by PCR after transfection with si-METTL3 and si-METTL14 into U266-R cells. (**E**) The expression of miR-98-5p was measured by PCR after transfection with si-METTL3 and si-METTL3 in U266-R cells. (**F**) The expression of circ_0000337 was measured by PCR in U266 cells over-expressing circ_0000337 with or without the m^6^A site mutated under actinomycin D (5 μg/mL) treatment for 0, 4, 8 h. **p<0.01.


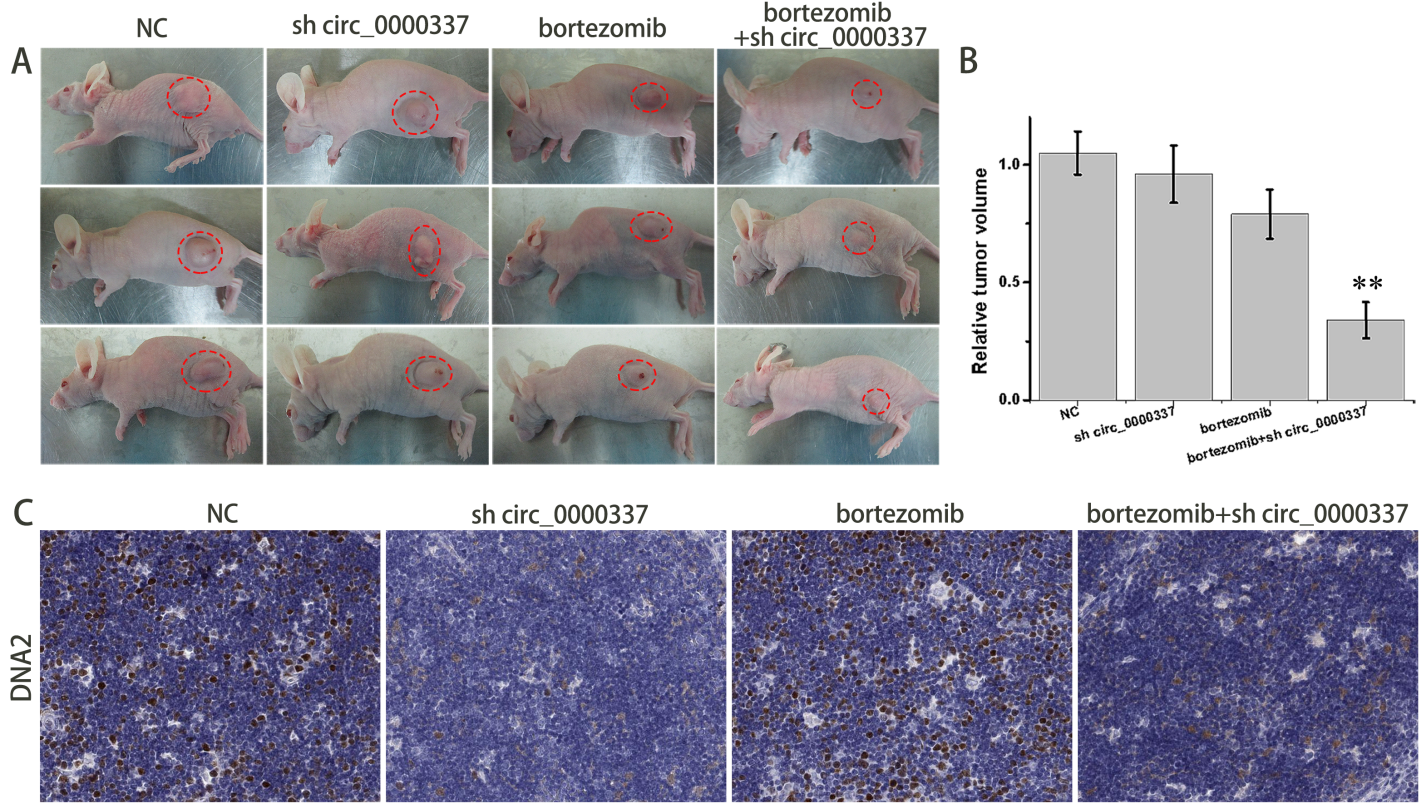


**Figure 9. In vivo targeting circ_0000337 reverses MM bortezomib resistance.** (**A**) Tumor images of nude mice in different treatment groups. A tumor-bearing mouse model was constructed by subcutaneous injection of U266-R cells. The lentivirus shcirc_0000337 was injected locally around the tumor and treated with bortezomib (0.5 mg/kg, intrabitoneal injection) for 2 weeks. (**B**) The relative volume of tumor in nude mice in different treatment groups. (**C**) DNA2 immunohistochemical staining (brown)of tumor tissue in different treatment groups. **p<0.01.
